# Olive tree at the intersection of environment, public health, and One Health: a sustainable path to global wellbeing

**DOI:** 10.3389/fpubh.2025.1658525

**Published:** 2025-09-19

**Authors:** Angeliki I. Katsafadou, Sofia I. Prodromou, Reza Aalizadeh, Jason C. White, Nikolaos S. Thomaidis, Ioannis S. Vizirianakis, Paul T. Anastas, Tassos C. Kyriakides, Harris Pastides, Prisco Piscitelli, Annamaria Colao, David C. Thompson, Vasilis Vasiliou

**Affiliations:** ^1^Department of Environmental Health Sciences, Yale School of Public Health, New Haven, CT, United States; ^2^Faculty of Public and One Health, University of Thessaly, Karditsa, Greece; ^3^Laboratory of Pharmacology, Faculty of Health Sciences, School of Pharmacy, Aristotle University of Thessaloniki, Thessaloniki, Greece; ^4^The Connecticut Agricultural Experiment Station, New Haven, CT, United States; ^5^Department of Chemistry, National and Kapodistrian University of Athens, Athens, Greece; ^6^Department of Health Sciences, School of Life and Health Sciences, University of Nicosia, Nicosia, Cyprus; ^7^Department of Biostatistics, Yale School of Public Health, New Haven, CT, United States; ^8^Department of Health Services Policy and Management, Arnold School of Public Health, University of South Carolina, Columbia, SC, United States; ^9^UNESCO Chair "Health and Sustainable Development", University of Naples "Federico II", Naples, Italy; ^10^Department of Psychology and Health Sciences, Pegaso Telematic University, Naples, Italy; ^11^Division of Endocrinology, Department of Clinical Medicine and Surgery, University of Naples "Federico II", Naples, Italy

**Keywords:** biodiversity, circular economy, environmental resilience, Mediterranean diet, nutrition, olive oil, olive by-products, One Health

## Abstract

The olive tree and its derivatives—olives, olive oil, and their by-products—are foundational to the Mediterranean diet and are increasingly recognized for their roles in nutrition, medicine, and ecological sustainability. Indeed, one of the most prominent examples of sustainable production and consumption paradigm in a changing climate lies in the olive sector, approached within One Health framework, i.e., the interconnectedness of human health with animal and environmental health. This review explores the multifaceted roles of olive cultivation, olive oil production and consumption, and olive by-products in relation to health benefits, sustainable agriculture, and environmental impact. Olive oil consumption offers significant human health benefits, primarily involving its anti-inflammatory and antioxidant properties. These effects, largely attributed to its rich composition of monounsaturated fatty acids and other antioxidants, mediate its cardioprotective and neuroprotective roles. Beyond human health, olive oil cultivation and its by-products (such as pomace and mill wastewater) have gained attention as valuable feed additives in animal nutrition. These enhance livestock health and welfare, improve meat and dairy quality, and promote sustainable agricultural practices and bioenergy production—ultimately reducing environmental impact and supporting circular economies. From an environmental perspective, the olive sector contributes meaningfully to soil conservation, biodiversity support, and climate change mitigation through carbon sequestration and reduced greenhouse gas emissions. As such, the olive tree is more than a source of a valuable food product: it is a nexus of sustainable development, public health, and ecosystem stewardship. Considering the olive sector within the One Health paradigm highlights its relevance in addressing global challenges at the intersection of food systems, health, and environmental sustainability.

## 1 Introduction

**Figure 1:**
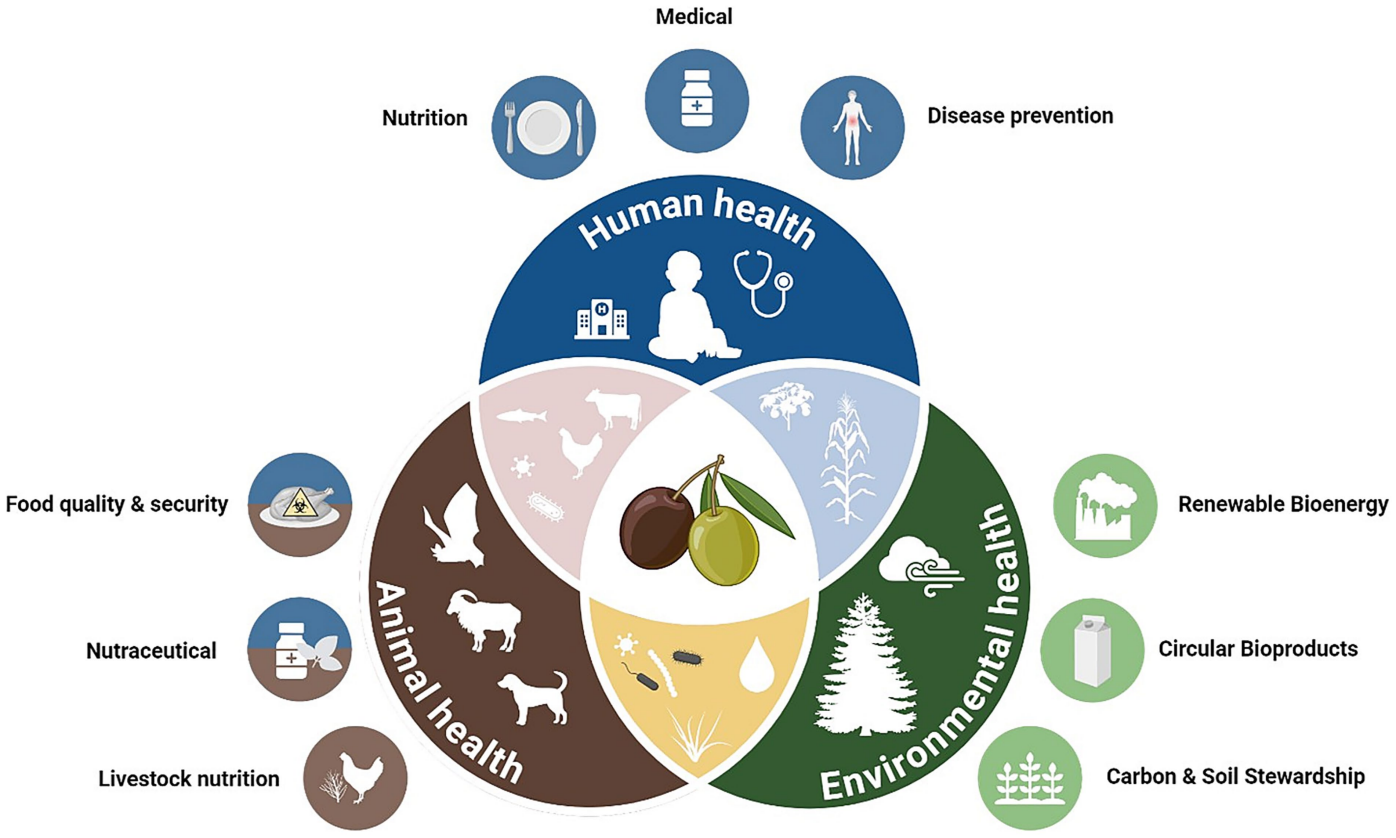
The One Health approach in the context of olives, olive oil and their by-products. The *blue* section highlights implications for human health, including nutrition, medical applications, and disease prevention. The *brown* section focuses on animal health, covering aspects such as livestock nutrition, nutraceuticals, food quality and security. The *green* section represents environmental health, including carbon and soil stewardship, circular bioproducts, and renewable bioenergy. The figure was created using BioRender (https://BioRender.com).

One Health is a multi-sectoral approach which recognizes that human health is connected to animal health and to the environment, emphasizing the need for integrated actions to address global health challenges (1, 2). Olives and olive oil, renowned for their nutritional and medicinal properties, represents a compelling case study within this framework due to its significant impacts across all three domains (3, 4) (Figure 1).

**Figure 2:**
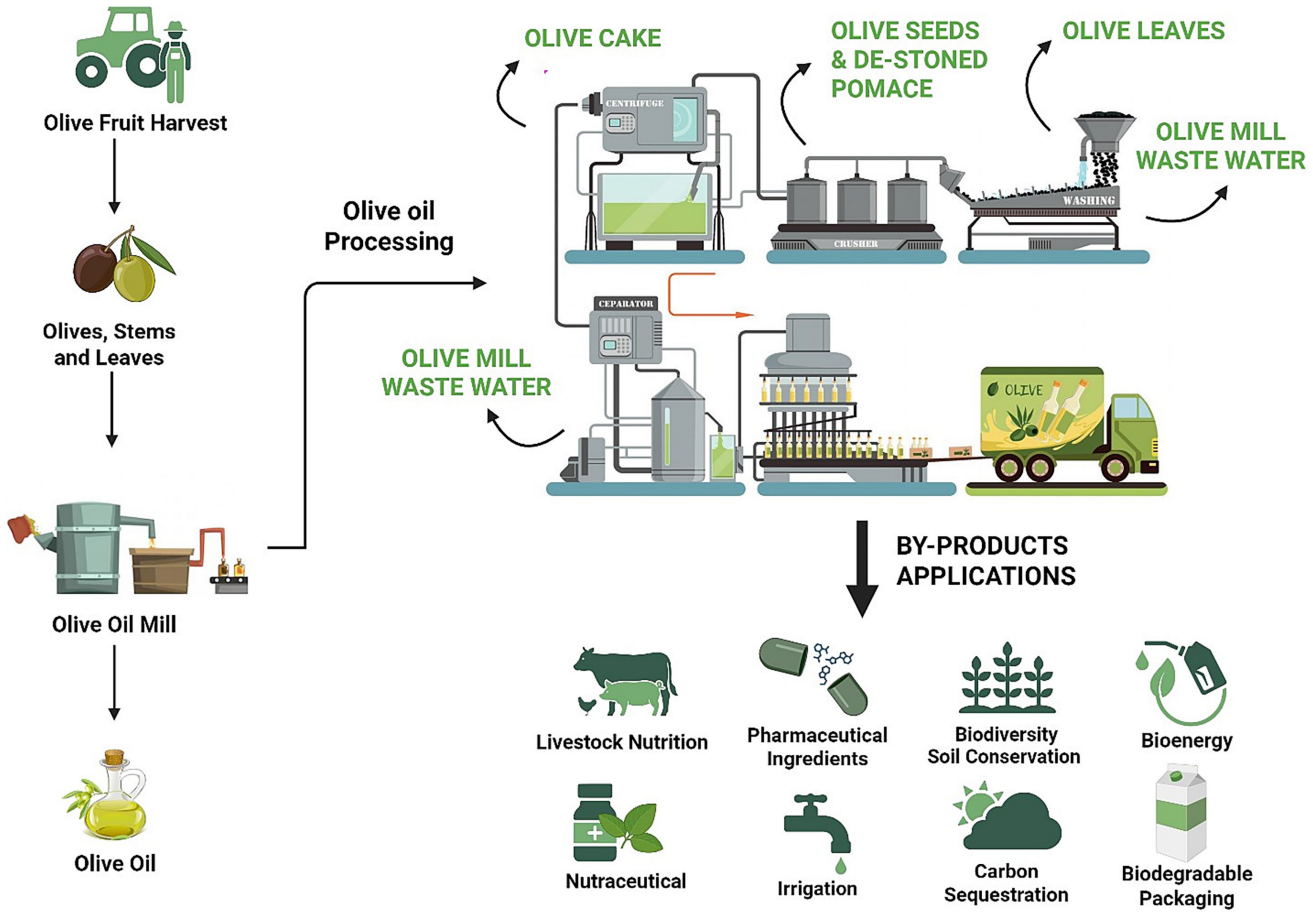
Olive oil production process and the utilization of its by-products. Olives (containing 10–25% Oil, 20–35% Dry Matter, 25–35% Pits, 65–75% Pulp and 45–55% Olive Water) are harvested and processed to produce olive oil. The resulting by-products—de-stoned pomace *(the by-product of olive oil extraction after the pit has been removed from the olive paste)*, olive mill wastewater, olive leaves, olive seeds, and olive cake *(the remaining pulp, skins, and sometimes stones after the oil has been extracted from the olives)*—are repurposed into a variety of applications, including livestock nutrition, nutraceuticals and pharmaceuticals, water irrigation, biodiversity and soil conservation, carbon sequestration, bioenergy production and biodegradable packaging. The figure was created using BioRender (https://BioRender.com).

As a staple of the Mediterranean diet, olives and olive oil has been celebrated for centuries for its role in promoting health and longevity, an acknowledgment reflected in its recognition by UNESCO as part of the Mediterranean diet’s Intangible Cultural Heritage of Humanity (5, 6). More recently, the concept has been expanded beyond the Mediterranean basin through the “Planeterranean” food-pyramid proposal for Asia, which adapts the diet’s sustainability and health principles to regional culinary traditions (7, 8). Rich in monounsaturated fats, polyphenols and antioxidants, olive oil consumption has been linked to up to a 31% reduction in cardiovascular events, a 28% lower risk of dementia-related death, and anti-inflammatory effects, including reductions in inflammatory markers, such as C-reactive protein and interleukin-6 (9–22). While most associations come from observational studies and should be interpreted with caution, evidence from intervention trials is growing. Some findings, especially in cognition, remain mixed, highlighting the need for more long-term randomized studies (16, 17). However, the relevance of olives and olive oil extends beyond human health. From an environmental perspective, the cultivation of olive trees plays an important role in promoting biodiversity, improving soil quality, and mitigating climate change through carbon sequestration (23, 24). In addition, by-products of olive oil production (such as pomace, leaves, pits and even mill wastewater) together represent up to 78% of the olive mass and are increasingly valorised in livestock feed. These by-products can replace 15–20% of conventional concentrate ruminant diets (the grain-based, high-energy component that complements bulkier forage), thereby closing resource loops within a circular One Health framework (Figure 2). This practice supports animal health, improves production efficiency, and reduces agricultural waste (25, 26), in accordance with circular economy principles and contributing to more sustainable agricultural systems (27, 28).

Considering olive cultivation in a One Health framework allows for a comprehensive assessment of the multifaceted contributions of olive tree and olives to human, animal, and environmental health. This integrated perspective highlights their potential not only as valuable nutritional and economic resources, but also as a crop that addresses critical global challenges related to sustainability, food security, and health. This review considers the role of olives, olive oil and their by-products within the One Health paradigm, and focuses on their health benefits, sustainable agricultural production practices and environmental implications of its production.

## 2 The health benefits of olives, olive oil and their by-products

The health-promoting properties of olives and olive oil are well-established, and supported by clinical, epidemiological and laboratory studies. They contain monounsaturated fatty acids (MUFAs), polyphenols, sterols and tocopherols which exhibit therapeutic potential for both humans and animals (11, 29–34).

### 2.1 Human health

Multiple studies have demonstrated the cardioprotective effects of olive oil, largely attributed to its rich content of MUFAs (primarily oleic acid) and a diverse array of bioactive compounds, including tocopherols, squalene, phytosterols, and various polyphenols (e.g., oleocanthal and oleuropein) (10, 11, 35, 36). These constituents help mitigate oxidative stress, inflammation, and lipid oxidation—key processes in the pathogenesis of atherosclerosis and other chronic diseases. Notably, phenolic alcohols (e.g., hydroxytyrosol, tyrosol), secoiridoids (e.g., oleuropein aglycone, oleacein, oleocanthal), lignans (e.g., (+)-pinoresinol, (+)-acetoxypinoresinol), and α-tocopherol have been identified as major contributors to the antioxidant and anti-inflammatory properties of extra virgin olive oil (EVOO) (3, 10, 35, 37–40). Reflecting these benefits, olive oil polyphenols (particularly hydroxytyrosol and its derivatives) have earned a health claim endorsement under EC Regulation 432/2012 (41). More recently, advances in machine learning and artificial intelligence (AI) have been applied to identify EVOO phytochemicals with the highest potential to modulate disease-associated protein networks, offering new opportunities for precision nutrition (42).

EVOO phenolics directly scavenge reactive oxygen species (ROS) (such as superoxide and hydroxyl and peroxyl radicals) through hydrogen atom donation. Compounds like hydroxytyrosol (HT) and oleuropein (OLE) also chelate transition metals (Fe^2+^, Cu^2+^), reducing oxidative damage (29, 43). Furthermore, these bioactives enhance endogenous antioxidant defense systems leading to increased expression of antioxidant enzymes, including superoxide dismutase (SOD), catalase, glutathione peroxidase (GPx), and heme oxygenase-1 (HO-1) (44, 45). In addition, EVOO compounds (e.g., OLE and HT) inhibit pro-oxidant enzymes [such as NADPH oxidase (NOX2/4) and xanthine oxidase], further reducing ROS generation (29, 46).

Olive oil constituents also modulate inflammation through several complementary pathways (47). Oleocanthal inhibits cyclooxygenase-1 and -2 (COX-1/COX-2), reducing prostaglandin synthesis in a manner similar to non-steroidal anti-inflammatory drugs (NSAIDs) (3, 48). Hydroxytyrosol and OLE suppress the NF-κB and AP-1 pathways, leading to reduced production of pro-inflammatory cytokines, including tumor necrosis factor alpha (TNF-α), interleukin 1β (IL-1β), and interleukin 6 (IL-6) (29, 43). In addition, HT has been found to interfere with the NLRP3 inflammatory complex, thereby limiting the activation of downstream inflammatory signals (49). Notably, a recent meta-analysis concluded that EVOO consumption did not consistently lower inflammatory markers (such as CRP or IL-6), reflecting variability in findings across intervention trials (50). The antioxidant and anti-inflammatory properties of EVOO translate into multiple cardiovascular health benefits (21). By reducing low-density lipoprotein (LDL) oxidation and enhancing high-density lipoprotein (HDL) functionality, EVOO constituents help prevent the accumulation of cholesterol-laden immune cells that drive atherosclerotic plaque development (10, 11, 36, 51). In hypercholesterolaemic subjects, higher nitric oxide (ΝΟ) bioavailability and lower oxidative stress after a high-phenolic EVOO meal improve endothelial-dependent vasodilation (ischemic reactive hyperaemia), an early protective mechanism against atherosclerosis (52). Moreover, longer-term olive oil interventions have been shown in multiple randomized controlled trials to increase brachial artery flow-mediated dilation, a marker of vascular health and predictor of reduced cardiovascular risk (53). Inhibition of platelet aggregation through lowering thromboxane A₂ production by oleocanthal supports anti-thrombotic effects (3, 48). Large clinical trials—most notably PREDIMED, a Spanish multicentre randomized controlled trial in 7,447 high-risk adults testing a Mediterranean diet supplemented with EVOO against a low-fat control—showed a significant reduction in rates of major cardiovascular events, indicating cardiovascular benefits linked to EVOO supplementation (11). Systematic reviews of similar interventions report modest improvements in standard lipid measures, such as LDL-C, HDL-C and triglycerides (54). In addition, a large meta-analysis of 33 randomized clinical trials found that EVOO consumption lowered fasting insulin and insulin resistance, as measured by the homeostasis model assessment of insulin resistance (HOMA-IR), but, interestingly, had no consistent effects on inflammatory markers (CRP, IL-6), lipids, or blood pressure (55). These findings suggest that while EVOO shows clear benefits for insulin sensitivity, its effects on inflammation and cardiometabolic risk factors remain heterogeneous across trials, which may help explain differences compared with individual studies reporting positive results.

EVOO has been associated with a lower risk of neurodegenerative diseases (including Alzheimer’s and Parkinson’s) (56–59) through multiple mechanisms. Preclinical and observational human studies suggest that phenolic components of olive oil (such as HT and oleacein)—may help preserve cognitive health in aging by modulating oxidative stress and inflammation; however, clinical trial evidence remains limited and inconsistent (60). Randomized clinical trials have reported improvements in memory performance and clinical dementia ratings in individuals with mild cognitive impairment following high-phenolic EVOO consumption (13). Activation of antioxidant enzymes (e.g., SOD and catalase) preserves mitochondrial integrity and promotes neuronal survival, a crucial effect given the overwhelming evidence implicating mitochondrial dysfunction as a causal factor in these diseases (29, 43, 58). EVOO secoiridoids (especially oleocanthal and oleacein) help strengthen the protective barriers between brain cells and reduce processes that can damage brain tissue (61, 62), maintaining blood–brain barrier function and preserving neuronal connectivity (13). Moreover, in Parkinson’s disease models, EVOO phenolics (HT, oleacein, mixed phenolic extracts) modulate microglial activation, thereby lowering IL-1β and TNF-α release and attenuating neuro-inflammation (63–65). Recent reviews suggest that dietary polyphenols (e.g., sulforaphane, resveratrol, luteolin, curcumin) improve oxidative stress and inflammation in autism models, alleviating impaired sociability and repetitive behaviors (66, 67). Small clinical studies have reported modest benefits in irritability and hyperactivity (68), although confirmatory trials are still lacking (66, 67, 69).

Epidemiological and clinical data indicate that regular EVOO consumption helps prevent and manage type 2 diabetes (12, 70, 71). In Mediterranean-diet cohorts, including the PREDIMED trial, higher EVOO intake was associated with markedly lower diabetes incidence compared to a low-fat control diet (12, 70). This finding is consistent with modest improvements in fasting glucose and insulin sensitivity observed in randomized EVOO trials (71, 72). Meta-analyses of Mediterranean diet adherence report an overall 16–19% risk reduction of diabetes (71, 72), and a recent dose–response meta-analysis that include cohort studies and randomized clinical trials found a 13–22% reduction in the risk of type 2 diabetes with daily olive oil consumption emphasizing the need for further randomized clinical trials to confirm causality (73). EVOO phenolics also display anti-cancer properties through multiple, complementary actions, including (i) repression of oxidative DNA damage, (ii) modulation of estrogen-receptor signaling, (iii) inhibition of pro-tumor inflammatory and angiogenic pathways, and (iv) promotion of tumor-cell apoptosis while blocking metastasis (29, 74–77). Population studies have linked higher olive oil intake to lower incidence of hormone-dependent malignancies, such as breast and ovarian cancer (78, 79). In addition, EVOO-derived compounds (like oleocanthal and OLE) have been shown to suppress proliferation across a range of tumor cell lines *in vitro* (80). Collectively, these anti-proliferative, pro-apoptotic and anti-angiogenic effects position EVOO polyphenols as plausible nutritional supplements for cancer prevention. While these findings are promising, most are derived from preclinical or observational studies. Clinical trials are needed to confirm whether these effects translate into consistent cancer risk reduction in humans. These mechanisms—particularly the modulation of oxidative stress and inflammatory pathways—are also relevant in animals, where olive-derived compounds demonstrate similar pathophysiological benefits.

### 2.2 Animal health

Olive oil, in combination with by-products from olive cultivation and processing (such as pomace, mill wastewater, leaves, and stones or seeds) is increasingly valued as a source of functional feed additives, due to their rich content of bioactive compounds, including polyphenols, MUFAs, sterols, and dietary fiber (25, 26, 81–87). These components offer antioxidant, antimicrobial, and anti-inflammatory properties, making them beneficial for animal nutrition and health, while contributing to sustainable waste management in olive oil production (86, 88–91).

In ruminants, incorporating olive oil by-products into feed has demonstrated notable nutritional and health benefits. For example, supplementation of diets with polyphenol-rich extracts from olive mill wastewater led to reductions in the urea content (up to 16%) and somatic cell counts (up to 59%) in Sarda ewes—findings indicative of improved udder health and reduced inflammation (92). In cattle, similar dietary inclusion of olive by-products (e.g., olive pomace) enhanced milk quality, increasing MUFA levels (≈5%) while reducing saturated fats (≈7%) (85, 93).

In swine, supplementing diets with HT- and polyphenol-rich olive by-products have been found to enhance immune function, reduce oxidative stress, and improve lipid metabolism (26, 86, 94). Studies indicate that HT not only supports antioxidant defense mechanisms but also mitigates inflammatory responses, which may contribute to better overall health and productivity in pigs (26, 95, 96). When destoned olive cake was included as 5–10% of the total feed, finishing pigs showed improved feed conversion ratios (FCR), reduced back-fat thickness and a healthier intramuscular fatty-acid profile, i.e., richer in MUFAs and polyunsaturated fatty acids (PUFAs) (97). In addition, supplementation of finishing diets with a polyphenol extract from olive mill wastewater positively remodeled gut microbiota and intestinal morphology, changes that support better gastrointestinal health (98).

In poultry production, the benefits of dietary supplementation with olive oil and its by-products have been well-documented, particularly for egg and meat quality (87, 99–102). Diets supplemented with 2–5% olive oil can lead to egg yolks with higher levels of the total unsaturated (mainly monounsaturated) fatty acid content (100). Similarly, the fortification of rations with 4–6% dried olive pulp deepened yolk color, reduced shell defects and positively modulated gut microbiota (103). Reductions in hens’ serum cholesterol have been reported, but evidence for direct cholesterol reduction in egg yolks remains inconclusive (100). Modification in the lipid composition of eggs is of particular interest given that eggs are a daily staple in many diets and, accordingly, even modest improvements in their nutrient profile could have a substantial public health impact. In broiler chickens, diets containing 2% or 4% olive cake meal (supplemented with *Bacillus licheniformis*) have been shown to improve weight gain and FCR, reflecting enhanced feed efficiency (104). Similarly, olive oil supplementation enhanced body weight gain, while olive cake inclusion (up to 15%) maintained feed intake and efficiency, with 10% boosting feed conversion and improving survival rates (105–107). Broilers given HT-rich olive by-products (e.g., olive mill wastewater permeate or polyphenol-rich EVOO) showed enhanced antioxidant status (as evidenced by higher catalase and superoxide-dismutase activities and lower lipid and protein oxidation in blood and tissues), while growth performance (body-weight gain and feed-conversion ratio) remained largely unchanged (108, 109). Incorporation of 5% olive oil in broiler diets improved the unsaturated-to-saturated fatty acid ratio in breast and drumstick meat while reducing serum triglyceride levels and increasing HDL cholesterol. However, early growth performance of the animals was slightly reduced (110). Overall, supplementation with olive oil and by-products in broilers enhances antioxidant status—an important benefit given that stressors common in commercial poultry production (environmental, pathogenic, and nutritional) negatively impact growth, health, and feed efficiency. However, the effects on growth performance remain inconsistent.

Beyond nutritional benefits, olive oil and its by-products enhance immune function and gut microbiota balance in livestock (26, 111). Enriched diets lower oxidative stress markers, improve animal health, and reduce antibiotic need, contributing to antimicrobial resistance mitigation (26, 86–89, 112, 113). Key phenolics (e.g., OLE and HT) modulate inflammatory and oxidative stress pathways, enhancing resilience (90). In broilers, dietary supplementation with olive oil increased antibody titers against Newcastle disease virus (105), while olive-derived supplements, up-regulated antioxidant enzymes (HO-1, SOD, catalase, GPx), and boosted IL-2/interferon-γ and IgA-IgG-IgM levels, changes that strengthen immune defenses (26, 83, 114). Inclusion of olive by-products in feed can also mitigate antimicrobial resistance by lowering caecal multidrug-resistant *Campylobacter* and ESBL (extended spectrum beta-lactamase)-producing *E. coli* loads and, *in vitro*, OLE and oleocanthal have been shown to inhibit bacterial efflux pumps and biofilm formation (115). Hydroxytyrosol has demonstrated antioxidant and immunomodulatory effects in immunosuppressed broiler chickens, by improving gut health, lowering inflammation, and strengthening important immune cells (e.g., CD4^+^ and CD8^+^ T-cells) (113). In swine, dietary supplementation with polyphenol-rich olive extracts fostered beneficial gut bacteria, suppressed pathogens, and promoted digestion and gut health (116, 117).

Feeding olive oil or its processing by-products to livestock consistently yields foods of animal origin with a more favorable lipid profile. In finishing pigs, replacing 5–10% of the concentrate portion of the diet with destoned olive cake increased the proportions of MUFA + PUFA in muscle without impairing growth performance (97). Dairy products also show improvements. For example, including olive cake in cow diets increased the oleic and conjugated linoleic acid content of cheese without affecting milk yield, and enhanced the appearance, aroma and flavor of the cheese (118). Similarly, when 8% olive cake was incorporated into Holstein cow rations, the resulting Provola cheese contained more oleic acid and retained bioactive polyphenols (119). In broiler chickens, adding 2.5–10% dried olive pulp produced breast meat richer in oleic acid and less prone to oxidation (101, 120). Collectively, these studies demonstrate that incorporating olive oil by-products into livestock feed can transform Mediterranean agro-waste into value-added pork, poultry, and dairy products with improved fatty-acid profiles and oxidative stability—traits that support their classification as functional foods. Beyond their nutritional value, olive-derived products also influence environmental dynamics—directly through agricultural practices and indirectly by shaping circular systems that impact soil, biodiversity, and climate resilience. These broader ecological roles are explored in the following section.

## 3 Impacts on environmental health and climate

The incorporation of olive oil by-products into animal feed provides both environmental and economic benefits (27). Olive oil production generates substantial waste, and repurposing these materials as livestock feed reduces pollution, supports sustainable waste management, and promotes circular economy principles (121, 122). By replacing a portion of conventional cereal-based feeds, olive by-products lower the ecological footprint of livestock production, and enhance resource efficiency (84, 85, 121, 123). Given that intensive agriculture consumes vast amounts of water, energy, and agrochemicals—accounting for ≈70% of global freshwater withdrawals (124, 125)—shifting feed sources away from high-input crops can yield measurable life-cycle savings and free water for human use. Anaerobic digestion of olive mill wastewater fosters also a circular economy by generating biogas and nutrient-rich digestate suitable for fertilization (126, 127), while olive seeds can be processed into functional protein isolates for food or feed applications, providing an additional high-value route for the valorisation of olive-mill solids (128, 129).

Olive trees, known for their longevity and adaptability, play a key role in Mediterranean agroecosystems by conserving soil, enhancing biodiversity, and supporting agroecological stability through their deep-root systems and low-input requirements (130, 131). Although well adapted to semi-arid climates, increasing exposure to prolonged droughts, temperature extremes and erratic weather are posing risks to olive trees yields and grove resilience (132).

These risks are projected to intensify under future climatic changes (particularly in the Mediterranean region), with possible negative consequences for the composition and nutritional quality of olive oil, as well as the sector’s long-term productive capacity (133). Paradoxically, these same shifts have expanded the geographical range of olive cultivation, enabling the crop to be established in regions previously considered unsuitable (133, 134). These considerations highlight the importance—but also the uncertainty—of future cultivation zones under evolving climate conditions. While efforts to develop heat- and drought-tolerant cultivars are showing promise (131, 133), field validation under real-world environmental variability remains limited. As such, climate adaptation strategies in the olive sector should be grounded in region-specific data, accounting for uncertainties in climate and yield projections, and informed by local agronomic knowledge (132, 133). Organic farming practices (such as reduced pesticide use and intercropping) promote biodiversity and soil health (135–137). Despite their potential, by-products of olive cultivation and harvesting are still infrequently used as fertilizer alternatives due to toxicity concerns (unless properly treated, such as through spray drying) (138). Composting or vermicomposting with bacterial and fungal communities is being widely investigated as a bioremediation step. These microbes metabolize phenolics, detoxifying the waste and rendering the resulting compost/vermicompost suitable for reuse as an organic soil amendment (135, 139). The utility/value of this approach was shown in a recent life-cycle study of organic olive-tree nurseries in Tuscany, where transitioning from conventional to organic practices (including the use of compost and reduced peat) reduced cradle-to-gate greenhouse gas emissions by 13%, rising to 15.7% when accounting for carbon stored in the seedlings (140).

While still in early development, emerging nanotechnologies (including metal-oxide nanofertilizers) present promising tools for reducing dependence on agrochemicals and enhancing nutrient-use efficiency in agriculture. These strategies have been tested in cereals and vegetables and may hold potential for improving crop resilience to abiotic stresses (e.g., drought, salinity) and biotic threats (e.g., pathogens) (141, 142). Notably, Zhao et al. (143) describe a suite of innovations—from stress-signaling primers to smart nutrient coatings—that collectively improve plant tolerance to drought, heat and pathogens. Cerium-oxide nanoclusters, for example, have been shown to activate abscisic acid (ABA)-responsive drought genes and boost biomass under water stress by ≈31%, demonstrating a substantive mitigation of water-stress damage (144). Similarly, seed priming with reactive oxygen species-generating nanoparticles has also improved antioxidant capacity and conferred multi-stress tolerance in maize (145). While these findings are compelling, their translation to perennial crops (such as olives) remain speculative. Olive-specific trials are lacking, and responses in woody plants may differ due to physiological and phenological differences. Nevertheless, early evidence from selenium-based nanomaterials have demonstrated the ability to enhance plant immunity and nutritional quality, suggesting future applicability in increasing olive resistance to fungal pathogens while enriching fruit micronutrient content (146). Additional studies are required to assess these technologies in olive-specific contexts and ensure safe, scalable use.

Olive trees help mitigate climate change through the process of carbon sequestration, both in their biomass and surrounding soil. As perennial plants, they absorb carbon dioxide over long time frames, with groves sequestering ≈2.2 metric tons of carbon per hectare per year (23, 147–149). Their extensive root systems help maintain soil organic carbon levels, further promoting long-term carbon storage (148, 150, 151). Emerging research suggests that olive trees could also play a role in improving environmental conditions in urban settings, such as air quality enhancement through pollutant capture (152).

Recently, the valorization of cellulose-rich olive oil pomace has gained attention for developing biodegradable food packaging materials as a sustainable alternative to plastics. Given its high cellulose and fiber content, pomace enhances the mechanical strength and water resistance of starch-based films, making them more suitable for food packaging applications (153). Olive stones are widely used as biomass fuel, particularly in Spain where they generate heat and electricity for agricultural operations and residential heating (154). In a recent innovation, Karim et al. developed a microwave-assisted hydrothermal carbonization process to convert olive pomace slurry into biochar-like hydrochar, a solid biofuel with high calorific value for electricity generation (155). Similar studies have shown that both hydrothermal carbonization and traditional slow-pyrolysis of olive residues yield carbon-rich biochar solids that can serve as renewable fuel, soil-amendment, and long-term carbon-sequestration agents, thereby extending the circular-economy benefits of the olive sector (126, 155, 156).

Of the many ways that the products of the olive sector bring benefit to humanity, perhaps one that is least developed and receives the least attention is the olive stone. This is likely due to the long tradition of thinking of biomass that contains high quantities of lignin as being recalcitrant and extremely difficult to process. In recent years, significant progress has been made in valorizing lignin (122, 157, 158). It has been estimated that the olive stone comprises 18–22% lignin (158). While lignin from olive stones has been demonstrated to be useful in applications ranging from biochar (159) to heavy-metal extractions from water (160), techniques are emerging to transform the stone lignin. For example, oxidative processes under relatively mild catalytic conditions allow the conversion of whole lignin into constituent specialty chemicals that can be used as high-value ingredients in formulated products, such as vanillin (3-methoxy-4-hydroxybenzaldehyde) and 2,6-dimethoxy-1,4-benzoquinone (DMBQ) (157). In addition, novel polycarbonate polymers can be formed by breaking down lignin into monomers and promoting subsequent repolymerization (161). The rapidly progressing research area of lignin processing holds promise for the olive stone to contribute to the overall economics of an olive refinery concept where every component adds value.

At the industry level, producers are increasingly adopting renewable energy sources (such as solar panels and wind turbines) that reduce reliance on fossil fuels and minimize emissions associated with production (162). The introduction and application of carbon-neutral initiatives (including reforestation projects, waste reduction strategies, and renewable energy integration) are also positioning the olive sector as a leader in climate-smart agriculture (27). These sustainability-driven efforts highlight a commitment to balancing productivity with environmental responsibility.

## 4 Conclusion

Olives, olive oil and their by-products play a pivotal role within the One Health framework, linking human health, animal nutrition, and environmental sustainability. As a keystone of the Mediterranean diet, the olive tree also reinforces sustainable food systems, linking cultural heritage, environmental stewardship, and long-term public health. The olive’s rich composition of monounsaturated fatty acids, polyphenols, and antioxidants provides significant cardioprotective, neuroprotective, and metabolic benefits, while its by-products enhance livestock health, improve food quality, and reduce agricultural waste. Olive cultivation supports biodiversity, soil conservation, and carbon sequestration, making it a sustainable agricultural practice. However, climate change and resource constraints still threaten the long-term viability of olive cultivation, necessitating renewable energy adoption, climate-resilient farming, and waste valorization. By embracing sustainable strategies and circular economy principles, the olive sector can continue to promote health, environmental stewardship, and economic resilience in a rapidly evolving global landscape.
